# The Testis-Specific Factor CTCFL Cooperates with the Protein Methyltransferase PRMT7 in *H19* Imprinting Control Region Methylation 

**DOI:** 10.1371/journal.pbio.0040355

**Published:** 2006-10-17

**Authors:** Petar Jelinic, Jean-Christophe Stehle, Phillip Shaw

**Affiliations:** Institute of Pathology, Centre Hospitalier Universitaire Vaudois, Lausanne, Switzerland; Harvard University, United States of America

## Abstract

Expression of imprinted genes is restricted to a single parental allele as a result of epigenetic regulation—DNA methylation and histone modifications. *Igf2/H19* is a reciprocally imprinted locus exhibiting paternal *Igf2* and maternal *H19* expression. Their expression is regulated by a paternally methylated imprinting control region (ICR) located between the two genes. Although the de novo DNA methyltransferases have been shown to be necessary for the establishment of ICR methylation, the mechanism by which they are targeted to the region remains unknown. We demonstrate that CTCFL/BORIS, a paralog of CTCF, is an ICR-binding protein expressed during embryonic male germ cell development, coinciding with the timing of ICR methylation. PRMT7, a protein arginine methyltransferase with which CTCFL interacts, is also expressed during embryonic testis development. Symmetrical dimethyl arginine 3 of histone H4, a modification catalyzed by PRMT7, accumulates in germ cells during this developmental period. This modified histone is also found enriched in both *H19* ICR and *Gtl2* differentially methylated region (DMR) chromatin of testis by chromatin immunoprecipitation (ChIP) analysis. In vitro studies demonstrate that CTCFL stimulates the histone-methyltransferase activity of PRMT7 via interactions with both histones and PRMT7. Finally, *H19* ICR methylation is demonstrated by nuclear co-injection of expression vectors encoding CTCFL, PRMT7, and the de novo DNA methyltransferases, Dnmt3a, -b and -L, in *Xenopus* oocytes. These results suggest that CTCFL and PRMT7 may play a role in male germline imprinted gene methylation.

## Abstract 2

The testes-specific factor CTCFL (also called BORIS) can cooperate with the arginine histone methyltransferase (PRMT7) to induce de novo DNA methylation at an imprinting control region.

## Introduction

Genomic imprinting is an epigenetic mechanism of transcriptional regulation that ensures restriction of expression of a subset of mammalian genes to a single parental allele. The *Igf2/H19* locus is the best studied example of imprinted gene regulation in which *Igf2* (insulin-like growth factor 2) is expressed uniquely from the paternal allele [1]. Control of *Igf2* expression is achieved by monoallelic methylation of an imprinting control region (ICR) located between the *Igf2* and *H19* genes [2]. The non-methylated ICR of the maternal allele functions as a chromatin insulator through interaction with the 11-zinc finger protein CTCF (CCCTC-binding factor) [2,3]. In contrast, CTCF cannot bind the methylated ICR of the paternal allele, and consequently, distally located enhancers can activate the *Igf2* promoter [2,3]. The CTCF protein is thus defined as a somatic regulator of imprinted gene expression [4].

*H19* ICR methylation is established during male germline development. At the outset of mouse testis development (12.5–13.5 days post coitum [dpc]), male ICR methylation is absent and is re-established during subsequent developmental stages (14.5–17.5 dpc) [5–7]. The de novo DNA methyltransferases, Dnmt3a and -L have been shown to play a key role in this initial ICR methylation [8,9], and their maximal expression coincides with these developmental stages [7,10]. No specificity of DNA binding is exhibited by the Dnmt3 subunits [11], and therefore it is thought that the de novo methyltransferases are recruited to sites of DNA methylation through interaction with specific chromatin modifications or a bridging protein(s) recognizing specific chromatin modifications.

A potential candidate for Dnmt3 recruitment could be a post-translationally modified histone(s). Histones are known to be subject to a large variety of modifications including methylation, acetylation, ubiquitination, and phosphorylation, each of which can occur at numerous residues, thereby contributing to histone structural diversity [12]. These modifications constitute the “histone code,” which can then be translated by interacting proteins into specific conformational alterations and/or DNA methylation [13]. The best example of this mechanism is recognition of trimethylated K9 histone H3 present in heterochromatic regions and subsequent Dnmt3 recruitment by HP1 (heterochromatin protein 1) [14].

A model for the acquisition of CpG methylation in ICR has been recently proposed [15]. The model invokes specific recognition of the ICR region that targets a histone modification, and subsequent recruitment directly or indirectly of the de novo DNA methyltransferases [15]. The only protein characterized to date to exhibit specific ICR recognition and binding is the ubiquitously expressed CTCF protein [2]. Recently, CTCFL/BORIS (CTCF like/brother of the regulator of imprinted sites; hereafter referred to as CTCFL), a testis-specific paralog of CTCF, has been characterized [16]. CTCFL possesses an 11-zinc finger region that is highly homologous to that of CTCF (74% identity), suggesting similar DNA recognition. The latter notion is supported by the demonstration of CTCFL binding in vitro to the FII element within the β-globin gene cluster, a characterized CTCF binding site [2,16]. The amino acid sequence flanking the zinc finger region of CTCFL exhibits no significant homology with CTCF, suggesting a function distinct from that of CTCF.

These characteristics thus render CTCFL an interesting candidate to participate in ICR methylation re-establishment. In the present report, we have pursued the possible role of CTCFL in the methylation of the *H19* ICR. Chromatin immunoprecipitation (ChIP) analysis indicates CTCFL association with two paternal ICRs, *H19* and *Gtl2*. Furthermore, the embryonic expression of CTCFL in the developing testis coincides with imprint re-establishment. Interestingly, the N-terminal region of CTCFL interacts with a protein methyltransferase, PRMT7, which methylates histones H2A and H4. Co-expression of CTCFL and PRMT7 and the de novo methyltransferases in *Xenopus* oocytes by microinjection of expression plasmids results in significant methylation of a co-injected ICR plasmid. Taken together, these results suggest that CTCFL may play a role in male germline *H19* ICR methylation.

## Results

### Expression of CTCFL during Testis Development

**Figure 1:**
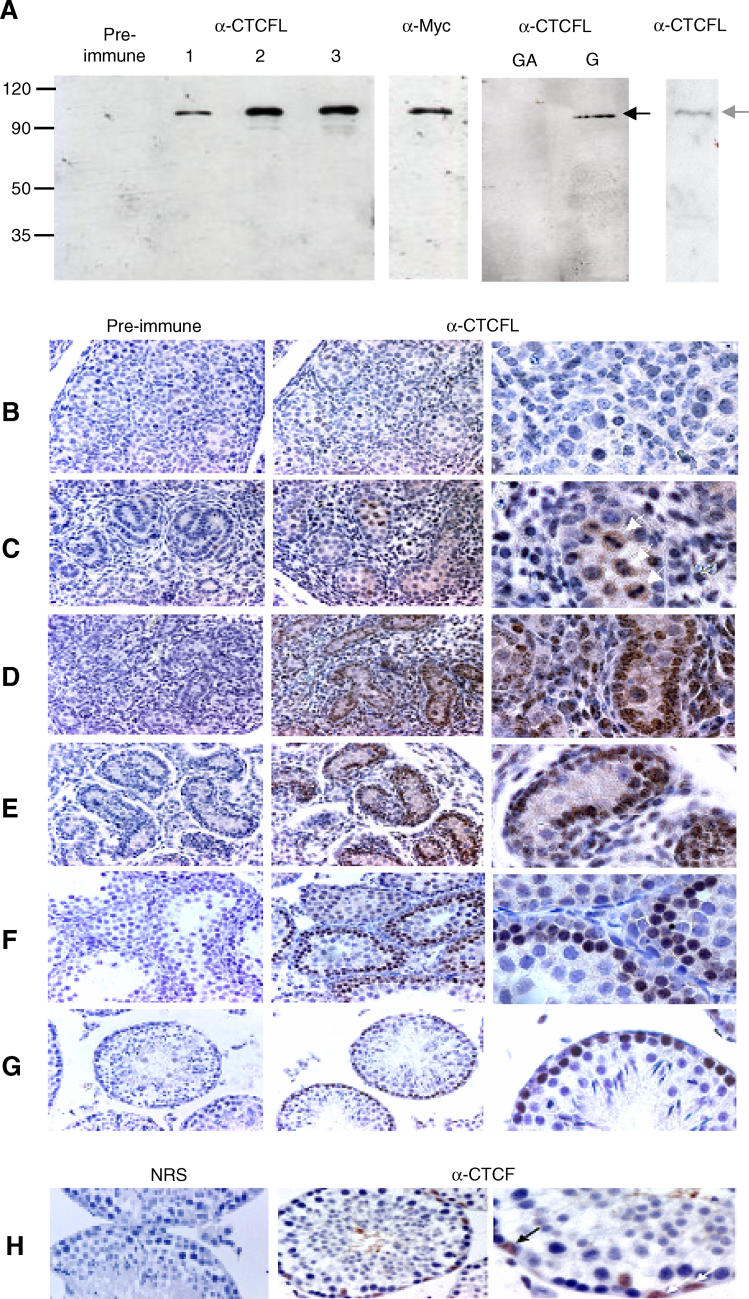
Immunohistochemistry of CTCFL Expression in Developing Testis (A) Characterization of α-CTCFL antibody. Myc-tagged CTCFL expressed in 293T cells was used to assess antibody titer and specificity. First (left) panel shows different anti-sera (dilution 1:1,000); pre-immune; 1: first bleed; 2: second bleed; 3: final bleed. Second panel: verification of myc-tagged CTCFL by α-myc antibody. Third panel: determination of α-CTCFL antibody specificity. The α-CTCFL antibody (dilution 1:10,000) was pre-incubated with GST-N-terminal CTCFL (GA) and GST (G). The antibody, pre-incubated with specific antigen, was neutralized, whereas α-CTCFL pre-incubated with GST alone retained its reactivity. Fourth panel: α-CTCFL reacted with a Western blot of adult testis extract. The black arrow indicates the position of myc epitope-tagged CTCFL, whereas the grey arrow indicates the position of endogenous CTCFL. (B–G) CTCFL expression in developing and adult testis. The pre-immune and adjacent α-CTCFL panels are at the same magnification (400×). A 5× magnification of the α-CTCFL image is given in the rightmost panel. (B) CTCFL is not detected at 13.5 dpc. (C) Mitotically arrested gonocytes (marked with white arrows) exhibit CTCFL staining at 14.5 dpc. (D) A few centrally localized gonocytes and cells at the periphery of seminiferous tubules express CTCFL at 17.5 dpc. (E) CTCFL is localized in gonocytes in newborn mice. (F) At 15 d after birth, nuclei of spermatogonia expressed CTCFL, as did their counterparts in adult testis (G). (H) Immunohistochemical detection of CTCF expression in adult testis. Normal rabbit serum (NRS) (left panel) and α-CTCF are presented as for (B–G). CTCF is expressed uniquely in Sertoli cells (marked with arrows).

Erasure and re-establishment of paternal ICR methylation takes place during embryonic testis development [5,6]. At 12.5 dpc, CpG methylation within the *H19* ICR has been erased in primordial germ cells (PGCs) and is subsequently re-established, such that by 17.5 dpc, significant *H19* ICR methylation is present [5,6]. To examine whether CTCFL expression coincides with the timing of re-establishment of the methylation, we performed immunohistochemical studies on embryonic and adult mouse testis using an α-CTCFL antibody (Figure 1). CTCFL was not detected at 13.5 dpc, but expression was observed in mitotically arrested gonocytes of 14.5-dpc embryos (Figure 1B and 1C). At 17.5 dpc, a few centrally located gonocytes and cells present at the periphery of the developing seminiferous tubules were positive (Figure 1D). Similar CTCFL localization was observed in newborn testis (Figure 1E). At 15 d after birth, nuclei of spermatogonia expressed CTCFL (Figure 1F), as did their counterparts in adult testis (Figure 1G). The developmental timing and cell-type commitment of CTCFL expression thus coincide with ongoing ICR methylation and parallel that of the de novo DNA methyltransferases.

Since CTCF and CTCFL were anticipated to exhibit similarity in DNA binding specificities [16], we also examined the cellular commitment to CTCF expression in adult testis (Figure 1H). Cells with triangular nuclei located along the basal lamina, consistent with the Sertoli cell phenotype, displayed nuclear staining. Thus, cell-type expression of CTCF is distinct from CTCFL in testis.

### CTCFL Binds Both the *H19* ICR and *Gtl2* Differentially Methylated Region In Vivo

**Figure 2:**
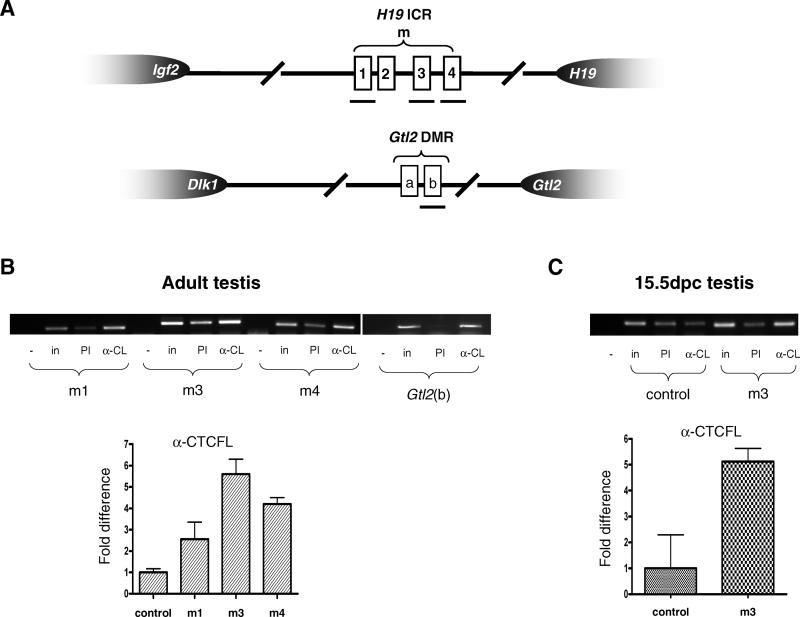
ChIP Analysis of CTCFL Association with the *H19* ICR and *Gtl2* DMR (A) Diagrams illustrate the regions within *H19* ICR and *Gtl2* DMR analyzed by ChIP. (B) Adult testis analysis. Upper panels show agarose gels of real-time PCR products for *H19* ICR and standard PCR products for *Gtl2* DMR. A no–DNA control (−)is indicated. Input (In) represents 10% of total amount of samples used. Pre-immune (PI) was used as a control for the enrichment of CTCFL immunoprecipitated with α-CTCFL (α-CL) antibody. For *Gtl2* DMR, normal rabbit serum (NRS) was used. Lower panel shows real-time PCR analysis of *H19* ICR. Control represents genomic region lacking CTCF binding consensus sequence. (C) The 15.5-dpc testis analysis. Figure is organized as for (B). In this case, control is included in the upper panel.

Given the high homology of the zinc finger regions of CTCF and CTCFL (74% identity), it is anticipated that the two proteins would recognize similar DNA sequences. CTCF has been shown to bind in vitro to the *H19* ICR in a methylation-sensitive fashion [17]. CTCFL, on the other hand, has been demonstrated to bind in vitro to the FII β-globin insulator, a characterized CTCF target [16], but specific ICR recognition by CTCFL has not been reported. Thus, we undertook an in vivo analysis of CTCFL association with the *H19* ICR by ChIP (Figure 2), using an α-CTCFL antibody and mouse testis chromatin (15.5-dpc and adult gonads). In addition, we analyzed CTCFL association with the differentially methylated region (DMR) of *Gtl2,* another characterized, paternally imprinted locus [18]. The mouse *Gtl2* DMR and the *H19* ICR contain two (a and b) and four (m1–m4) CTCF consensus binding sites, respectively [2,19]. Chromatin immunoprecipitation of 15.5-dpc testis by the α-CTCFL antibody yielded a significant enrichment of the m3 region of the *H19* ICR (Figure 2C). Similar enrichment of the m1, m3, and m4 regions of the *H19* ICR was observed in adult testis samples (Figure 2B). The analysis of the b region of the *Gtl2* DMR in adult testis also appears enriched relative to normal rabbit serum controls (Figure 2B). The *Gtl2* locus ChIP was not evaluated by quantitative PCR. A control genomic region without CTCF consensus sites was negative for enhancement. This control sequence is a unique sequence containing three CpGs with a GC content of 49% located on mouse Chromosome 8.

These results demonstrate that CTCFL specifically associates in vivo with both the *H19* ICR and the *Gtl2* DMR in testis.

### CTCFL Interacts with the Protein Methyltransferase PRMT7

The amino acid sequences N-terminal to the zinc finger regions of CTCFL and CTCF do not exhibit significant homology [16], suggesting different functions, possibly by association with distinct proteins. To identify potential interacting proteins, CTCFL was used as bait in a yeast two-hybrid screen with a mouse testis cDNA prey library. Thirteen different mRNAs were present in a total of 33 colonies. We retained a recently described protein arginine methyltransferase PRMT7 [20,21] and a novel arginine-rich testis-specific histone H2A variant, observed two and seven times, respectively.

**Figure 3:**
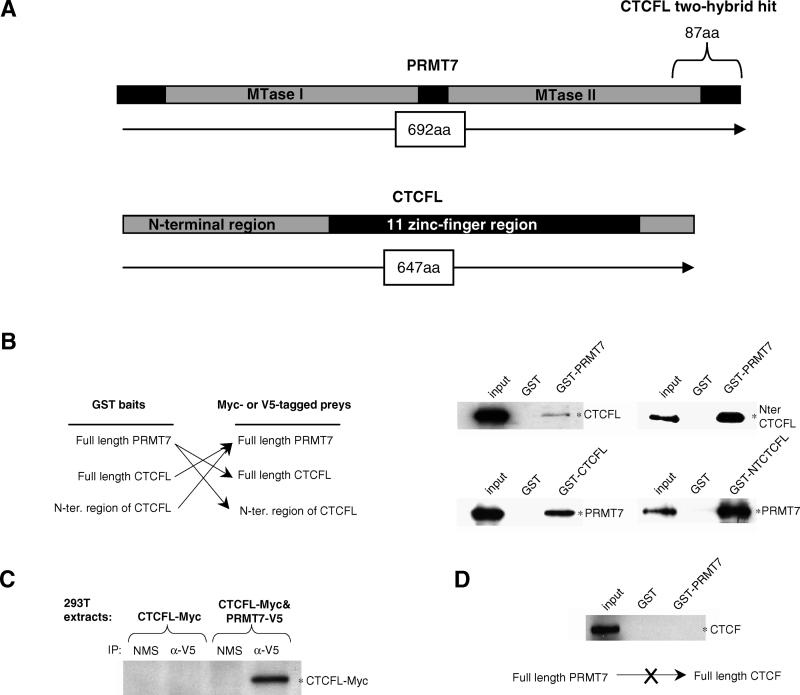
N-terminal CTCFL Interacts with PRMT7 (A) Diagrams show the domain structure of the PRMT7 and CTCFL proteins. MTase I and II represent the methyltransferase regions within PRMT7. The position of the recovered yeast two-hybrid clone of PRMT7 is indicated. (B) Reciprocal GST pull-downs of CTCFL and PRMT7. Scheme of the tested interactions is given in diagrams. GST and GST fusion proteins (CMV-GST vector) were co-expressed in 293T cells with myc- or V5-tagged preys. N-ter., N-terminal. (C) Co-immunoprecipitation of CTCFL and PRMT7. The 293T extracts containing over-expressed CTCFL-Myc or CTCFL-Myc&PRMT7-V5 were immunoprecipitated with α-V5 antibody or normal mouse serum (NMS). CTCFL was detected with α-Myc antibody. N-ter., N-terminal. (D) No interaction between CTCF and PRMT7 is detectable by GST pull-downs.

The cDNA sequence within PRMT7 prey clones encodes the carboxy-terminal 87 amino acids (aa) of the 692–aa protein, downstream of two predicted methyltransferase domains (Figure 3A). To validate this putative interaction, a CMV-GST (cytomegalovirus–glutathione S-transferase) PRMT7 (full length) was co-expressed in 293T cells with epitope-tagged CTCFL (N-terminal region and full length). Lysates were prepared and GST fusion proteins purified with glutathione beads. CTCFL was detected by Western blotting (Figure 3B). A reciprocal assay with CMV-GST-CTCFL (N-terminal region and full length) and epitope-tagged PRMT7 was also performed. Both assays verified interaction between the N-terminal region of CTCFL and full-length PRMT7 in 293T cells (Figure 3B). The N-terminal region of CTCFL used in these experiments corresponds to the aa sequence preceding the zinc finger region, which is approximately 255 aa in length. Further evidence of CTCFL-PRMT7 interaction is provided by the presence of CTCFL in the immunoprecipitate of co-expressed PRMT7 (Figure 3C). An interaction assay with CTCF (CMV-GST-PRMT7 and epitope-tagged CTCF) did not detect any association with PRMT7 (Figure 3D).

### CTCFL Interacts with Histones H1, H2A, and H3

**Figure 4:**
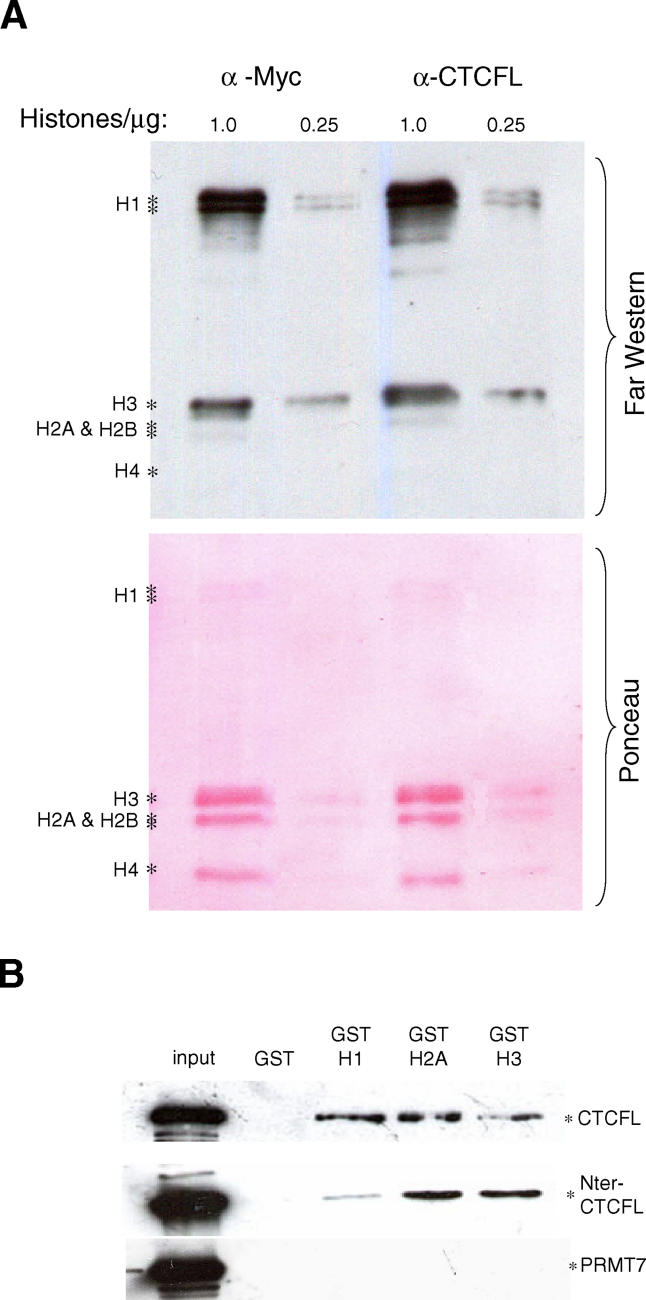
CTCFL Interacts with Histones H1, H2A, and H3 (A) Farwestern analysis. Western blots containing 1-μg and 0.25-μg histones were reacted with transfected 293T cell lysates expressing myc-tagged CTCFL. The presence of CTCFL was detected by α-Myc and α-CTCFL antibodies. Individual histones are indicated. The lower panel shows the ponceau red–stained Western blot. The migration of the individual histones are indicated to the left. (B) N-terminal and full-length CTCFL interacts with histones H1, H2A, and H3. Bacterial-produced GST histone fusion proteins were reacted with lysates from 293T cells transfected with either full-length or N-terminal CTCFL expression vectors. No detectable interaction of PRMT7 with histones is observed.

As mentioned in the previous section, a second CTCFL interaction candidate identified in the yeast two-hybrid screen was a novel testis-specific histone H2A variant. This result prompted us to examine the possibility that CTCFL also interacts with other canonical histones. CTCFL association with histones was initially examined by Farwestern analysis, which detected histones H1 and H3 (Figure 4A). Recognition of histones H1 and H3, and H2A by CTCFL was further examined by GST pull-down assays. Histones H1, H3, and H2A interacted with both full-length and N-terminal CTCFL (Figure 4B). No CTCFL was detected in control GST reactions. No detectable PRMT7 was observed in parallel interaction assays with histones (Figure 4B).

### PRMT7 Is Expressed in Germ Cells during Testis Development

**Figure 5:**
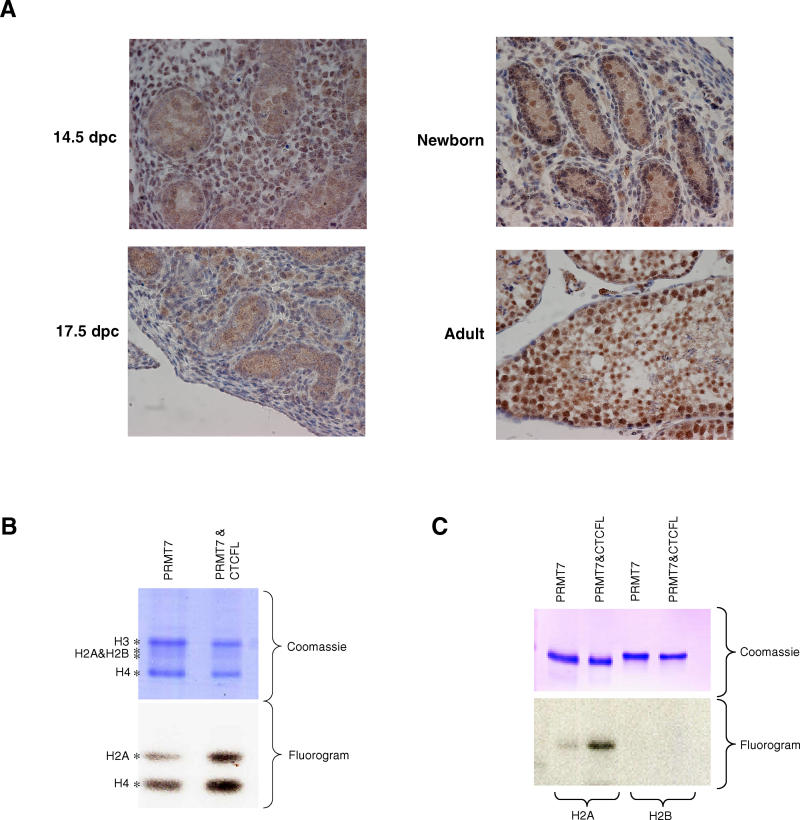
Developmental Expression and Methyltransferase Activity of PRMT7 (A) Developmental expression of PRMT7. Immunohistochemistry with α-PRMT7 on testis at the indicated stages of development. (B) PRMT7 in vitro methyltransferase reactions with total histones were performed either with α-V5–immunopurified PRMT7-V5 or α-V5–co-immunopurified PRMT7-V5&CTCFL-Myc. Upper panel shows Coomassie staining. Lower panel shows fluorogram (7-d exposure). Methylation of histones H2A and H4 is stimulated by co-immunoprecipitating CTCFL. (C) Discrimination between H2A and H2B methylation. For these histone methyltransferase reactions, CTCFL-myc and PRMT7-V5 were immunopurified separately using α-Myc and α-V5 antibodies, respectively, in reactions containing either H2A or H2B. Upper panel shows Coomassie staining. Lower panel shows fluorogram (7-d exposure). Stimulation of histone H2A methylation is observed with the addition of immuno-purified CTCFL.

Given the interaction of CTCFL and PRMT7, it was appropriate to evaluate the developmental expression of PRMT7 in the developing testis to determine whether or not it coincides with that of CTCFL. Immunohistochemistry with α-PRMT7 evidenced the expression of PRMT7 in all stages examined (Figure 5A). All cells within the developing tubule are positive, including gonocytes and spermatogonia that were positive for CTCFL expression. The expression of PRMT7 in all cell types present in testis is consistent with its ubiquitous tissue expression (expressed sequence tags [ESTs] can be found in the UniGene database [http://www.ncbi.nlm.nih.gov/entrez/query.fcgi?db=unigene&term=Mm.251804] as Mm.251804).

### CTCFL Stimulates PRMT7 Methylation of Histones H2A and H4 In Vitro

PRMT7 has been recently shown to catalyze arginine methylation of histones H2A and H4 [21]. PRMT7 expressed in and purified from bacteria exhibited little to no activity on several protein substrates, including histones H2A and H4 [20,21]. However, PRMT7 immunopurified from transfected HeLa cells exhibited significant activity in methyltransferase reactions [21]. A possible explanation for this increased activity is a post-translational modification of PRMT7 or the presence of an accessory protein(s) in HeLa cells [21]. It has been shown that CTCFL is constitutively expressed in HeLa cells [16], raising the possibility that CTCFL may function as an accessory protein of PRMT7.

To test this hypothesis, we used immunopurified CTCFL and PRMT7 for in vitro methyltransferase assays. Initially, either V5-immunopurified PRMT7 alone or V5 co-immunopurified PRMT7 and CTCFL-myc were used for methyltransferase assays with total histones (Figure 5B). Methylation of histones H2A and/or H2B and H4 was observed. Enhancement of methylation was observed when CTCFL had been co-immunopurified with PRMT7. To distinguish between histones H2A and H2B, separate reactions were performed (Figure 5C). For these reactions, CTCFL-Myc and PRMT7-V5 were independently immunopurified with α-Myc and α-V5 antibodies, respectively, and subsequently eluted with corresponding epitope-tagged peptides. Only histone H2A methylation was observed. Enhanced methylation of histones H2A and H4 by PRMT7, upon addition of immunopurified CTCFL, supports the idea that CTCFL functions as an accessory protein for PRMT7 activity.

### CTCFL Directs *H19* ICR Methylation in *Xenopus* Oocyte Injection System

Thus far we have shown that the developmental expression profile of CTCFL is coincident with re-establishment of imprints. In addition, CTCFL is bound to *H19* ICR in vivo, interacts with PRMT7 and histones H1, H2A, and H3 and stimulates H2A and H4 methylation by PRMT7. The discovery of these molecular interactions prompted us to address the question as to whether or not CTCFL and PRMT7 could participate in specific ICR methylation with the de novo DNA methyltransferases, Dnmt3a, Dnmt3b, and Dnmt3L. To test this hypothesis, we co-injected cDNA expression vectors and ICR plasmids into the nucleus of stage VI oocytes of *Xenopus*. The advantages of this assay are numerous. There is an abundant reserve of histones, which are capable of packaging injected DNAs into chromatin [22]. Oocytes lack male germline–specific factors. Imprinting does not occur in *Xenopus* [23]. In addition, one can analyze individual oocytes injected with a small quantity of ICR (40 pg). Co-injection of a GFP (green fluorescent protein) expression plasmid facilitates the identification of successfully injected oocytes.

Following DNA injection and oocyte incubation, GFP-positive oocytes were selected by fluorescence microscopy. DNA was then extracted and treated with sodium bisulfite, to determine CpG methylation. Subsequently, the m3 *H19* ICR region was amplified by PCR with specifically designed primers, then cloned and sequenced. Controls for DNA contamination were carried out for each series of injected oocytes by analyzing non-injected oocytes in parallel.

**Figure 6:**
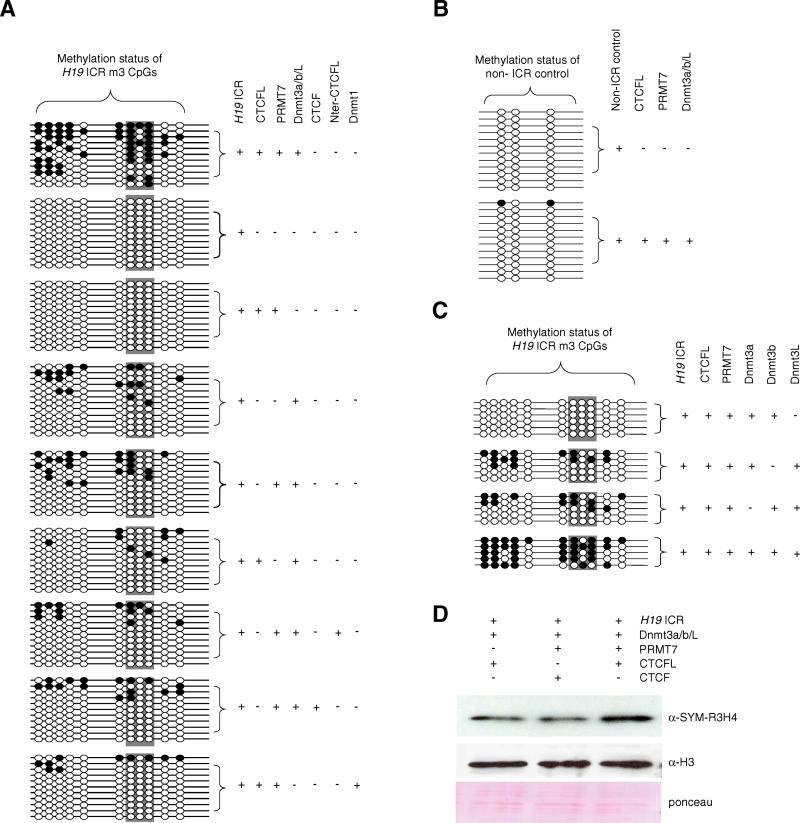
Analysis of De Novo *H19* ICR Methylation by Nuclear Injection of Expression Plasmids into *Xenopus* Oocytes The experiment tests the ability of combinatorial expression of proteins encoded by the expression plasmids to methylate co-injected *H19* ICR. A *H19* ICR plasmid was co-injected with variety of cDNA expression plasmids; GFP fluorescence was used to select oocytes that had been properly injected. Efficiency of bisulfite modification was determined by evaluating transformation to T of non-CpG Cs. The maximum non-transformed Cs observed was one out of 51. (A) The specific expression plasmids co-injected with *H19* ICR are indicated on the right side of the figure. Left panels indicate the methylation status of the 11 CpGs within the m3 region of the *H19* ICR. Grey-shaded parts indicate the position of the CTCF consensus sequence within m3. Open circle (○) indictates non-methylated CpG; filled circle (•) indicates methylated CpG. Optimal methylation is dependent on the expression of CTCFL, PRMT7, and all Dnmt3s. Oocytes were analyzed 48 h after injection. (B) Methylation analysis of co-injected control DNA plasmid**.** Organization of presented data is as for (A). The control fragment contains a unique sequence fragment present on Chromosome 8 and was also used as a control in ChIP analysis (Figure 2B and 2C). No significant methylation was observed with expression of CTCFL, PRMT7, and the Dnmt3a, -b, and -L, confirming sequence specificity of the ICR methylation analyzed in (A). (C) Functional analysis of the Dnmt3 isoforms. Organization of presented data is as for (A). Omission of Dnmt3L reduces methylation to 0%. Removal of either Dnmt3a or Dnmt3b significantly reduced observed methylation. Maximal ICR methylation is observed when all three Dnmt3s are co-injected with CTCFL and PRMT7. Oocytes were analyzed 72 h after injection. (D) Detection of SYM-R3H4 in sulfuric acid–extracted proteins from microinjected *Xenopus* oocytes. Expression plasmids injected are indicated on the figure. An increase in SYM-R3H4 is observed in the presence of both CTCFL and PRMT7. The middle panel shows reaction with an α-H3 antibody as a control for histone loading, whereas the lower panel shows ponceau red staining.

When CTCFL, PRMT7, Dnmt3a, Dnmt3b, and Dnmt3L expression plasmids were co-injected with *H19* ICR, a significant fraction of the CpGs in the m3 region of the *H19* ICR were methylated after 48 h of incubation (Figure 6A). In contrast, no CpG methylation was observed when the *H19* ICR alone was injected, indicating the absence of DNA methylation by endogenous factors in the *Xenopus* oocyte. Co-injection of CTCFL and PRMT7 expression vectors with ICR did not yield any CpG methylation, suggesting that endogenous Dnmt3 activity is lacking or insufficient. We then proceeded with injection of various combinations of the expression plasmids to assess the requirement of each corresponding protein in the observed ICR DNA methylation. Oocytes injected with Dnmt3a, Dnmt3b, and Dnmt3L, and *H19* ICR yielded few methylated CpGs (Figure 6A), demonstrating that co-expression with CTCFL and PRMT7 is crucial for efficient ICR methylation, and that over-expression of all three Dnmt3s alone gives rise to a low level of methylation [24]. Low methylation levels were also observed when either CTCFL or PRMT7 were co-injected with Dnmt3s (Figure 6A). This result further underscores the need for both CTCFL and PRMT7 in conjunction with the Dnmt3s to achieve significant ICR methylation. The sequence specificity of observed DNA methylation was assessed by co-injecting a control plasmid, without CTCF binding sites (ChIP control in Figure 2), along with CTCFL, PRMT7, and Dnmt3s expression plasmids (Figure 6B). Only low levels of CpG methylation were observed when the control plasmid was injected, confirming the sequence specificity of CTCFL-mediated ICR methylation. Control experiments, replacing the CTCFL expression plasmid with one encoding CTCF, did not yield significant ICR methylation (Figure 6A). Taken together, these results demonstrate that expression of CTCFL, PRMT7, and Dnmt3s in *Xenopus* oocytes results in specific and efficient methylation of co-injected *H19* ICR.

Having demonstrated the value of the *Xenopus* system to reflect the contribution of individual proteins on specific CpG methylation, we next evaluated the contribution of each Dnmt3 isoform in *H19* ICR methylation (Figure 6C). Omission of either Dnmt3a or Dnmt3b resulted in intermediate methylation levels after 72 h of incubation. This observation suggests that both Dnmt3a and Dnmt3b can participate in ICR methylation in the *Xenopus* system. When Dnmt3L was omitted from injected expression plasmids, no ICR CpG methylation was observed, highlighting its essential role. Replacing the Dnmt3 expression plasmids by one encoding Dnmt1 does not result in significant ICR methylation, consistent with a specific requirement for the de novo methyltransferases (Figure 6A). Positive controls, with injection of all expression plasmids and ICR, yielded higher levels of CpG methylation after 72 h of incubation than that observed after 48 h.

### Expression of PRMT7 and CTCFL in *Xenopus* Oocytes Yields Detectable Arginine 3 Methylation of Histone H4

Having shown that PRMT7 methylates histones H2A and H4 in vitro, we wished to determine whether or not this modification occurs in oocytes expressing PRMT7 and CTCFL. Given the high copy number of ICR plasmid injected into oocytes during the above experiments (approximately 10^6^), we reasoned that if PRMT7 in association with CTCFL methylates R3 of histone H4, it would be detectable by analysis of total oocyte histones. To test this idea, histones were extracted from oocytes with sulfuric acid and the resulting Western blots were reacted with α-SYM-R3H4 antibody (directed against a GR_Me2(sym)_GKGGKG peptide) for detection of SYM-R3H4 (symmetrical dimethyl arginine 3 histone H4; Figure 6D). When ICR was co-injected with expression plasmids for both PRMT7 and CTCFL, an increase in SYM-R3H4 was observed. However, if the CTCFL expression plasmid was replaced with a CTCF expression plasmid or if the PRMT7 expression plasmid was omitted, the level of detectable SYM-R3H4 is lower. An α-histone H3 was used as a control for histone loading. No signal was detected when an α-asymmetrical dimethyl arginine H4 R3 antibody was used to probe the same Western blot (unpublished data). The observed increase in SYM-R3H4 in total oocyte histones suggests that PRMT7, in collaboration with CTCFL, methylates at least histone H4, consistent with in vitro observations (Figure 5).

### Developmental Accumulation of SYM-R3H4 Parallels ICR Methylation

**Figure 7:**
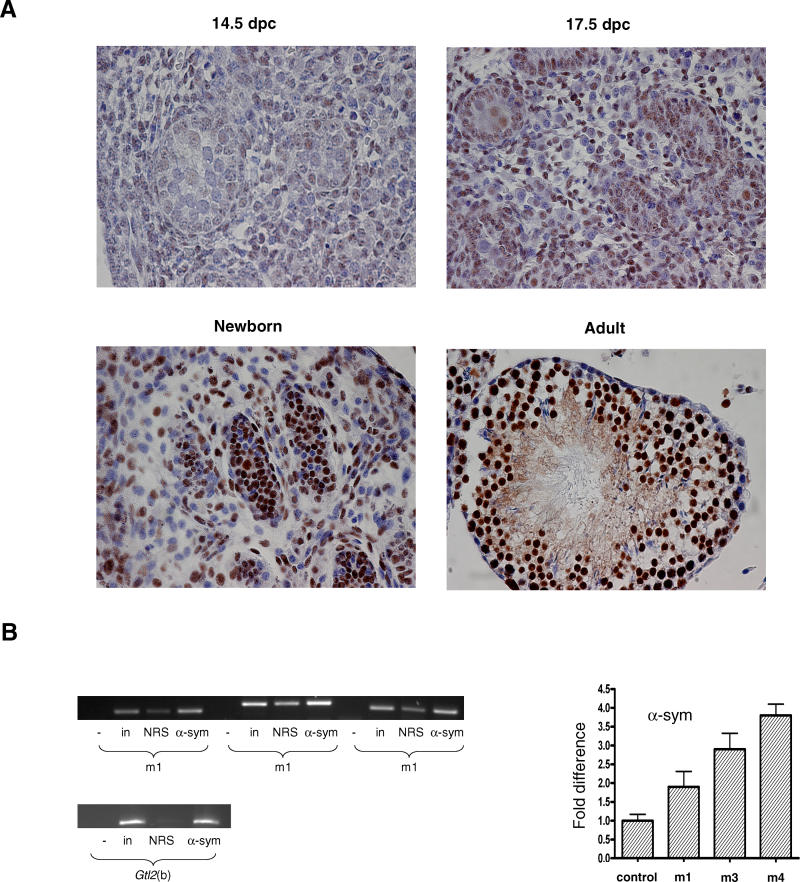
Developmental Accumulation of SYM-R3H4 in Mouse Testis (A) Significant SYM-R3H4 is first observed in the nuclei of cells composing the testis at 17.5 dpc. Subsequent stages, newborn and adult, exhibit strong nuclear staining of the majority of cells within the testis. (B) ChIP analysis of SYM-R3H4 in adult testis. The right panel shows the quantitative PCR analysis of the m1, m3, and m4 regions whereas the left panel shows an ethidium bromide–stained gel of the PCR products. Also shown are the PCR products of the ChIP analysis of the b region of *Gtl2* DMR.

If SYM-R3H4 is functioning to signal recruitment of the de novo DNA methyltransferases, then one would anticipate developmental accumulation of this modification in testis, paralleling ICR methylation. Immunohistochemistry with α-SYM-R3H4 antibody yields detectable nuclear staining at 17.5 dpc (Figure 7A). Subsequent developmental stages evidence a progressively stronger signal up to adult stages. The spermatogonia within the adult testis are positive for SYM-R3H4. There is a slight lag in the detection of SYM-R3H4 in testis, relative to the CTCFL expression (17.5 dpc vs. 14.5 dpc), consistent with a modification resulting from a process being initiated around 14.5 dpc.

A ChIP analysis with α-SYM-R3H4 on adult testis chromatin evidenced a significant enrichment of this histone modification at the m1, m3, and m4 regions of the *H19* ICR (Figure 7B), relative to a control genomic region. Analysis of the b region of the *Gtl2* DMR also evidenced the presence of this modification.

## Discussion

The present characterization of CTCFL, a CTCF paralog, supports its involvement in male germline imprinting. ChIP analysis demonstrates that CTCFL is associated in vivo with the *H19* ICR at 15.5 dpc. The binding of common regions by CTCF and CTCFL is consistent with the high homology between their zinc finger domains (74% identity, 84% positivity). The mutually exclusive expression in testis excludes competition for binding sites. CTCFL is detected in the male germline during testis development shortly after the erasure of methylation marks on ICR (14.5 dpc), and coincides with maximal expression of the de novo DNA methyltransferases, Dnmt3a and Dnmt3L [7,25]. The expression of CTCFL during embryonic development and its association with the *H19* ICR would suggest that it associates with non-methylated ICR.

The observed interaction of CTCFL with PRMT7, and histones H1, H2A, and H3 provides additional insight into its role in imprinting. PRMT7 has been reported to methylate arginine residues of histones H2A and H4 in vitro [21], and more precise analysis indicated a symmetrical dimethylation of arginine residues [21]. Non-exhaustive experiments with peptide substrates indicate that PRMT7 methylates a glycine-arginine-glycine motif [20], which is present in the N-terminal region of histones H2A and H4. The absence of detectable interaction of PRMT7 and histones (Figure 4B) and the stimulation of in vitro methylation by CTCFL (Figure 5) suggests that PRMT7 may rely upon CTCFL for efficient association with histones. The latter could be accomplished in a specific chromatin region due to the DNA binding specificity of CTCFL. The expression of PRMT7 in the embryonic testis is consistent with a role in *H19* ICR methylation.

Interaction between CTCFL and histones is of relevance from several viewpoints. The reported nucleosome positioning at the *H19* ICR places CTCF consensus sequences in the inter-nucleosome linker region [25]. Histone H1 and the N-terminal tail of histone H3 have been shown to stabilize chromatin structure [26], suggesting that their interaction with CTCFL may stabilize CTCFL's association with ICR. CTCFL interaction with histone H2A, a PRMT7 substrate, has particularly interesting functional implications. Attempts to detect association of H2A with PRMT7 were unsuccessful (Figure 4B), raising the possibility that CTCFL simultaneously interacts with histone H2A and PRMT7, thus presenting PRMT7 with its histone substrate for methylation. This notion is supported by the observed stimulation of PRMT7-mediated methylation of histone H2A by CTCFL (Figure 5B and 5C). The present results on PRMT7 and its association with CTCFL are analogous to observations made on other PRMTs [27,28]. PRMT5 has been shown, for example, to form a complex with the hSWI/SNF chromatin remodelers BRG and BRM, and this association enhances PRMT5 methyltransferase activity [28]. PRMT1 interacts with the transcription factor YY1 and is thereby targeted to specific promoters, where it catalyses adjacent histone H4 methylation in vivo [29]. This arginine methylation has been shown to be essential for activation of promoters to which YY1 is bound [30]. These observations open the possibility that the PRMT7-dependent increase in SYM-R3H4 observed in *Xenopus* oocytes is occurring on nucleosomal histones adjacent to the *H19* ICR, when CTCFL is expressed. Results of SYM-R3H4 ChIP analysis with testis chromatin are supportive of this interpretation.

A consideration of the experiments carried out in *Xenopus* oocytes should take into account the number of ICR plasmid copies present. Forty picograms of injected ICR plasmid corresponds to approximately 10^6^ copies. This elevated copy number probably renders the system less sensitive to endogenous components. The ICR methylation observed in the oocyte experiments is occurring on a significant fraction of this large number of injected ICR plasmid molecules. Secondly, the dependence on the injected expression plasmids for methylation to occur is probably due to the large number of templates. This does not rule out that endogenous factors may participate, yet the primary players in the reaction are likely to be those encoded by the co-injected expression plasmids.

Symmetrical dimethylation of R3 in histone H4 by PRMT7 was observed to increase when PRMT7 and CTCFL are co-expressed along with ICR in *Xenopus* oocytes. This augmentation is dependent on both proteins in that no increase in SYM-R3H4 methylation is observed when CTCF is expressed in place of CTCFL. Furthermore, omission of PRMT7 also evidences no increase in R3H4 methylation. In addition, the progressive accumulation of SYM-R3H4 in developing testis parallels expression of CTCFL and PRMT7. The dependence of ICR methylation in *Xenopus* oocyte experiments on co-expression of PRMT7 and CTCFL and the observed in vitro interaction of CTCFL with histones are consistent with such activity. The results of *Xenopus* oocyte microinjections indicate that expression of both proteins is necessary for efficient *H19* ICR methylation. In addition, ChIP analysis of adult testis indicated that SYM-R3H4 is present within *H19* ICR chromatin. The sequence specificity of the observed ICR methylation is supported by the lack of significant methylation when a control plasmid lacking CTCF consensus sequences was injected in place of *H19* ICR (Figure 6B).

Knockout mouse models of Dnmt3a and Dnmt3L have shown that both are necessary for appropriate maternal and paternal imprinting [8,9]. Results obtained with the heterologous *Xenopus* oocyte system are consistent with these observations. No CpG methylation was observed when Dnmt3L was omitted from the injected expression plasmids (Figure 6C). In contrast, Dnmt3b knockout mice do not exhibit any deficiencies in ICR methylation in the male germline [8], suggesting that expression of Dnmt3b is not critical for this event. Nonetheless, examination of Dnmt3b RNA/protein expression during the developmental interval of ICR methylation indicates that there is significant expression of a splice variant, Dnmt3b2, in testis from 14.5dpc to 17.5dpc [25,31]. Thus, Dnmt3b may participate in ICR methylation, but is not absolutely essential. Our results would be supportive of such a view. Dnmt1 expression is unable to compensate for the de novo methyltransferase expression, supportive of the specificity of ICR methylation in *Xenopus* oocyte experiments (Figure 6 ).

Several recent reports have indicated that the mutation of the four characterized CTCF binding sites, m1–m4, does not alter the establishment of *H19* ICR imprints in the male germline or the non-methylated status of the ICR in the female germline [32,33]. The created mutations were demonstrated in vitro to drastically alter CTCF binding. However, no in vivo confirmation of altered CTCF binding was presented, leaving open the possibility that alternative binding sites for CTCF exist within the 1.8-kilobase ICR sequence. In the present report, we demonstrate in vivo binding of CTCFL to the *H19* ICR, however, we have not investigated the precise sequence to which it binds. In order to reconcile the CTCF binding site mutant studies with the present results, one must consider the possibility that the sequence recognition of CTCFL is similar to, but distinct, from that of CTCF. Closer inspection of the sequence homology of the individual zinc fingers is consistent with such an interpretation. Two zinc fingers (fingers 1 and 11) of CTCFL show no significant homology with CTCF (two-sequence alignment), whereas the other fingers range from 54% to 95% amino acid homology (average, 67% identity).

A question that arises from the present results concerns the nature of recruitment of the Dnmt3s to *H19* ICR. Clearly the de novo DNA methyltransferases are efficiently recruited, given the level of specific methylation observed, yet the mechanism remains undefined. A possible explanation may come from recent studies on the PWWP domain. The observation that the PWWP domain shares similarities in both sequence and structure with the Tudor domain [34,35], which recognizes symmetrical dimethylated arginines [36,37], opens the possibility that dimethyl arginine–containing histones would be capable of recruiting the Dnmt3a and Dnmt3b subunits through interaction with their PWWP domains. Alternatively, there is a bridging protein(s) present in *Xenopus* oocytes that is responsible for the recognition of the dimethyl arginine histones, and interaction with this protein(s) recruits the Dnmt3s.

A recent publication by Vatolin et al. describes partial demethylation of the MAGE-A1 promoter either following treatment of cells with 5-aza-2′-deoxycytidine or upon over-expression of CTCFL [38]. However, neither the mechanism of demethylation nor the role of CTCFL in the demethylation was examined.

**Figure 8:**
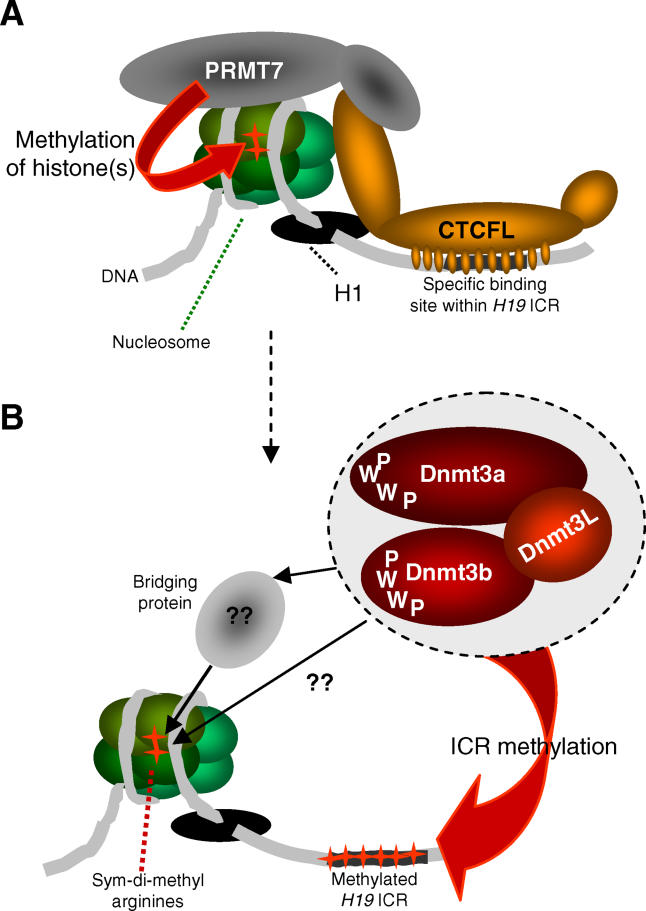
Model of *H19* ICR Methylation (A) An initial complex of CTCFL and PRMT7 localizes to the *H19* ICR, resulting in symmetrical dimethyl modification of arginine residues in histones H2A and H4 composing the adjacent nucleosomes. The localization to ICR is assured by the zinc finger portion of CTCFL, whereas the interactions with histones and PRMT7 are taking place via the N-terminal portion of the protein. (B) Following disengagement of the CTCFL complex, the de novo DNA methyltransferases Dnmt3a and Dnmt3b are recruited by either their PWWP motifs or through a bridging protein to the methylated histones. Dnmt3L is recruited by direct interaction with Dnmt3a and Dnmt3b. Subsequent to their recruitment, the de novo DNA methyltransferases methylate adjacent CpGs, resulting in ICR methylation.

In an effort to integrate the individual observations of protein interactions and the observed ICR methylation in *Xenopus* injection experiments, we propose a model for ICR methylation (Figure 8). The initial event is recognition and binding of the ICR chromatin by the zinc finger region of CTCFL, which then recruits PRMT7 to the chromatin site by interaction via its N-terminal region. Subsequently, PRMT7 catalyses the methylation of arginine residues in the GRG motif present in adjacent histones H2A and H4. The latter event is stimulated by the ICR-bound CTCFL scaffold. Following disengagement of the CTCFL-PRMT7 complex, the de novo DNA methyltransferases are recruited to the ICR chromatin region, possibly through recognition of dimethylated arginine residues by the PWWP domains of Dnmt3a and Dnmt3b or by a bridging protein provided by the oocyte.

## Materials and Methods

### α-CTCFL antibody preparation and characterization.

Immunization of rabbits with GST N-terminal CTCFL (1–229 aa) fusion protein was done in collaboration with Eurogentec (Seraing, Belgium). The fusion protein was expressed in Bl21 bacteria and purified using standard protocols. Use of the antibody in both immunoprecipitation and ChIP is described in the relevant sections. Characterization of the anti-sera is presented in Figure 1A.

### ChIP analysis.

ChIP was performed according to the Upstate Biotechnology's protocol (Upstate Biotechnology, Waltham, Massachusetts, United States) with minor changes. Adult mouse testes and embryonic gonads were homogenized in 5 ml and 1 ml of phosphate-buffered saline (PBS), respectively, and fixed with formaldehyde (1% v/v) for 10 min at room temperature. The reaction was stopped with 125 mM glycine. After washing twice with PBS, the cells were lysed with 1-ml SDS-lysis buffer (1% SDS, 10 mM EDTA, 50 mM Tris-HCl [pH 8.1]) for 15 min on ice and subsequently sonicated 2 × 45 s at 30% power (SONOPLUS Ultrasonic homogenizers HD 2070; Bandelin Electronics, Berlin, Germany) to obtain an average DNA length of 500 nt. In the case of 15.5-dpc testis, chromatin was reduced in size by digestion with restriction enzymes, according to Murrell et al. [39]. The sample was centrifuged at 20,000×*g* for 15 min, and the chromatin supernatant was diluted 10-fold with dilution buffer (0.01% SDS, 1.1% Triton X-100, 1.2 mM EDTA, 16.7 mM Tris [pH 8.1], 167 mM NaCl, 11% glycerol) and stored at −80 °C. After pre-clearing, 1.5 ml of prepared chromatin (approximately one third of testis) was incubated overnight at 4 °C with 40-μl salmon sperm DNA/protein A agarose slurry and 10 μl of either α-CTCFL or pre-immune serum. The samples were washed with 1 ml of each buffer and eluted twice with 250-μl elution buffer. NaCl was added (final concentration 300 mM), and the samples were incubated at 65 °C for 5 h to reverse crosslinking, and subsequently treated with proteinase K mix (final concentration: 40 μg ml^−1^ proteinase K, 40 mM Tris-HCl [pH 6.5], 10 mM EDTA) at 45 °C for an additional 2 h. After extraction with phenol/chloroform, the samples were precipitated with ethanol plus glycogen carrier. DNA was recovered in 50 μl of H_2_O. Real-time PCR analyses were carried out on all *H19* ICR ChIP samples. Optimization of reactions were carried out with all primer probe sets. Further, linearity of primer probe sets was verified by DNA dilution series over 3 logs. Standard curves of input DNA were carried out with all PCR analyses of ChIP. Primer and Taqman probe sequences are available on request. The analysis of the real-time PCR of ChIPs was according to Litt et al. [40].

### Immunohistochemistry and Western blot analysis.

α-CTCFL antibody and pre-immune sera were used at 1:1,000 dilution for all immunohistochemistry. α-CTCF IgG fraction (#06–917; Upstate cell signaling, Millipore, Billerica, Massachusetts, United States) and normal rabbit IgG fractions were used at 1:100 dilutions. Standard epitope retrieval methods were used for detection of both CTCFL, PRMT7, SYM-R3H4, and CTCF [41]. Secondary antibody used was Envision from DakoCytomation (Glostrup, Denmark). α-SYM-R3H4 antibody (ab5823; Abcam, Cambridge, United Kingdom), α-asymmetrical dimethyl arginine 3 H4 (07–213; Upstate, Millipore), and α-H3 (ab1791, Abcam) were used at the dilutions indicated by the suppliers for Western blot analysis.

### Yeast two-hybrid screen.

A yeast two-hybrid screen was performed with the Matchmaker GAL4 Two-Hybrid System 3 (Clontech, Palo Alto, California, United States) according to the manufacturer's instructions. A mouse testis cDNA library was prepared using a library construction kit (Clontech). All screening was carried out with four drop-out selection media. Isolated candidates were further screened for β-galactosidase activity. DNA was prepared from prey candidates by standard procedures and amplified by PCR with primers flanking the cloning site. Each insert was sequenced from the 5′ end to allow identification by BLAST and to verify retention of the upstream open reading frame. All selected prey candidates were re-transformed into bait strains to verify interactions.

### Vector construction.

GST fusion clones of PRMT7, CTCFL, and N-terminal CTCFL (1–229 aa) were constructed in a CMV-GST eukaryotic expression vector [42]. Myc-tagged CTCFL, and V5-tagged PRMT7, CTCF, N-terminal CTCFL, and Dnmt3L clones were ligated into the EF-1a eukaryotic expression vector (Invitrogen, Carlsbad, California, United States). Both CMV-GST fusion and Myc- or V5-tagged expression vectors were transfected into the 293T cells with CaPO_4_ [43]. Dnmt3a and Dnmt3b expression constructs were kind gift of F. Fuks and T. Kouzarides. All expression constructs used in *Xenopus* oocyte injections were confirmed by Western blot analysis with lysates from transfected 293T cells. GST fusion histones H1, H2A, and H3 were constructed in pGEX vectors and expressed in Escherichia coli Bl21 strain. Details of vector construction are available on request.

### Protein extraction.

Transfected 293T cells were washed twice in cold PBS and then resuspended in 1 ml (per 10-cm^3^ dish) of lysis buffer (150 mM NaCl, 50 mM Tris-HCl [pH 7.5], 10% glycerol, protease inhibitors: PMSF and Complete from Roche). After sonication (4 × 5 s at 25% power), Triton X-100 was added to a final concentration of 0.5%, and samples were centrifuged at 20,000×*g* for 15 min, and the supernatant was transferred to separate tubes. Myc- and V5-tagged protein lysates were snap-frozen and stored at −80 °C until use. CMV-GST fusion bait proteins, after extraction, were further purified. A total of 50 μl of glutathione-sepharose beads (G-4510; Sigma, St. Louis, Missouri, United States) were added to GST fusion protein lysates and incubated for 2 h at 4 °C. The immobilized GST fusion proteins were washed four times in lysis buffer plus 0.5% Triton X-100 and stored at 4 °C until use. A similar protocol of extraction and purification was followed for GST fusion proteins expressed in bacteria with different sonication conditions (5 × 10 s at 50% power). Histones were extracted from *Xenopus* oocytes by incubation on ice with in 0.4 N H_2_SO_4_ for 1 h. Proteins in the resulting supernatant were precipitated with ten volumes of acetone overnight at −20 °C.

### Protein interaction assays.

GST pull-downs with fusion proteins expressed in bacteria were performed according to standard procedures [44]. Co-transfection of CMV-GST fusion (bait) and epitope-tagged (prey) constructs in 293T cells are referred to as in situ GST interaction assays. In situ GST pull-down reactions were done overnight at 4 °C. In all assays, following the interactions, beads were washed 4 times in lysis buffer and boiled in 2× SDS loading buffer and analyzed by Western blotting.

### Histone methyltransferase reactions.

Immunoprecipitation of V5-tagged PRMT7 and myc-tagged CTCFL were according to standard procedures using protein G beads. The reactions with separate histones H2A and H2B (Roche, Basel, Switzerland) were done as follows. V5- or myc- immunopurified PRMT7 and CTCFL were eluted using epitope peptides V5 and myc, respectively. Equivalent amounts of PRMT7 were used in histone methyltransferase reactions [21] with or without CTCFL using C^14^ methyl S-adenosyl methionine (Hartmann Analytic, Braunschweig, Germany). Reactions were incubated overnight at 30 °C. Following the reactions, proteins were separated on 15% PAGE, embedded with 18% sodium salicylate, dried, and then exposed for fluorography. PRMT7 and CTCFL were co-immunoprecipitated using α-V5 antibody for methyltransferase reactions with total histones (Roche).

### Nuclear microinjection into *Xenopus* oocytes.

Nuclear microinjection into stage VI oocytes was done as previously described [45] with minor modifications. Injections into the nucleus were performed without prior centrifugation. The DNAs injected per oocyte were: 40 pg of target DNA (ICR or non-ICR), 200 pg of each expression plasmid with CMV or EF-1a promoters (CTCFL, PRMT7, DnmT3a, DnmT3b, and DnmT3L) and 200-pg pEGFP-C1 vector (Clontech). Injected oocytes were incubated at 19 °C for 48 or 72 h. The total amount of plasmid DNA injected thus depended upon the precise experiment carried out, yet gave a total amount of 1,240 pg maximally when six expression plasmids were injected. No carrier plasmid was used to normalize total injected plasmid DNA between experiments. Western analysis of Dnmt3a and Dnmt3b expression indicated no perturbation of expression of either protein when expressed alone or together (Figure S1).

### DNA isolation, bisulfite modification, and sequencing

DNA was isolated from GFP-positive oocytes by incubation in STOP buffer (1% SDS, 30 mM EDTA, 20 mM Tris-HCl [pH 7.5], 500 μg ml^−1^ Proteinase K) for 2 h at 37 °C. Subsequently the DNA was extracted twice with phenol/chloroform and ethanol precipitated. DNA was recovered in H_2_O, mixed with 2-μg salmon sperm DNA, and digested with the restriction enzyme ScaI (Roche) for 2 h at 37 °C, subsequently phenol/chloroform extracted, ethanol precipitated, and resuspended in 50-μl H_2_O. Bisulfite treatment of DNA was based on previously described method [46]. DNA was denatured by adding 5.5 μl of freshly prepared 3 M NaOH and incubating at 95 °C for 3 min, and immediately placed on ice. A total of 500 μl of freshly prepared Na-bisulfite mix (2.85 M Na-bisulfite, 2.7 mM hydroquinone, and 400 mM NaOH) was added and placed in PCR machine for bisulfite transformation (2 h at 55 °C, 1 min at 95 °C, 1 h at 55°C, 1 min at 95 °C, and 1 h at 55 °C). The samples were desulfonated on columns from QIAquick PCR purification kit (Qiagen, Valencia, California, United States) and purified with buffers from the same kit as follows. Five volumes of PB buffer was added to samples, and the mixture was applied to columns and washed with PE buffer. Samples were desulfonated with NaOH/ethanol (150 mM/90%) at room temperature for 10 min, washed with PE, and eluted in 50-μl EB buffer (10 mM Tris-HCl [pH 8.5]) for 15 min at 70 °C. A total of 5 μl of bisulfite-transformed DNA was amplified in 50-μl PCR reactions (40 cycles; annealing temperature was 50 °C) using corresponding meth-primers. (Primer sequences are available upon request.) PCR products were gel purified, cloned into pGEM-T vector, and sequenced with BigDye Terminator v1.1 Cycle Sequencing Kit (Applied Biosystems, Foster City, California, United States) in ABI 3100 sequencer. Efficiency of bisulfite modification was determined by evaluating transformation to T of non CpG Cs. The maximum non-transformed Cs observed was one out of 51.

## Supporting Information

Figure S1 Western Blot Analysis of Dnmt3a and Dnmt3b Expressed in Nuclear-Injected *Xenopus* Oocytes Left and right panels show analysis of Dnmt3a and Dnmt3b, respectively. Nuclear injections into *Xenopus* oocytes: 3a (GFP&Dnmt3a), 3a+T (GFP, Dnmt3a, Dnmt3L, ICR, and CTCFL&PRMT7), 3a/b (GFP and Dnmt3a&Dnmt3b), 3a/b+T (GFP, Dnmt3a, Dnmt3b, Dnmt3L, ICR, and CTCFL&PRMT7), 3b (GFP&Dnmt3b), and 3b+T (GFP, Dnmt3b, Dnmt3L, ICR, and CTCFL&PRMT7).There is no change in expression of either protein when expressed alone or together. Note, the panels showing GFP expression in the samples 3a/b and 3a/b+T are identical.
(2.6 MB PPT)

### Accession Numbers

GenBank (http://www.ncbi.nlm.nih.gov/Genbank) accession numbers for the genes discussed in this paper are mouse *CTCF* (NM_181322), mouse *CTCFL* (DQ153171), mouse Dlk1/Gtl2 DMR (AJ320506), mouse *Dnmt1* (BC053047), mouse *Dnmt3a* (NM_007872), mouse *Dnmt3b* (NM_001003961), mouse *Dnmt3L* (NM_019448), mouse IGF2/h19 ICR (AY849916), and mouse *PRMT7* (AY673972).

## Abbreviations

aa: amino acid
ChIP: chromatin immunoprecipitation
DMR: differentially methylated region
dpc: days post coitum
ICR: imprinting control region
